# Machine Learning–Enhanced Surveillance for Surgical Site Infections in Patients Undergoing Colon Surgery: Model Development and Evaluation Study

**DOI:** 10.2196/75121

**Published:** 2025-10-01

**Authors:** Ugur Celik, Feifan Liu, Kimiyoshi Kobayashi, Richard T Ellison III, Yurima Guilarte-Walker, Deborah Ann Mack, Qiming Shi, Adrian Zai

**Affiliations:** 1 Center for Clinical and Translational Sciences University of Massachusetts Chan Medical School Worcester, MA United States; 2 Department of Population and Quantitative Health Sciences Division of Health Informatics and Implementation Science University of Massachusetts Chan Medical School Worcester, MA United States; 3 Department of Medicine University of Massachusetts Chan Medical School Worcester, MA United States; 4 UMass Memorial Medical Center Worcester United States; 5 Infection Control Department UMass Memorial Medical Center Worcester, MA United States

**Keywords:** surgical site infection, machine learning, surveillance, electronic health records, natural language processing, colon surgery, risk prediction, Extreme Gradient Boosting, XGBoost, random forest, artificial intelligence, AI

## Abstract

**Background:**

Surgical site infections (SSIs) are one of the most common health care–associated infections, accounting for nearly 20% of all health care–associated infections in hospitalized patients. SSIs are associated with longer hospital stays, increased readmission rates, higher health care costs, and a mortality rate twice that of patients without infections.

**Objective:**

This study aimed to develop and evaluate machine learning (ML) models for augmenting SSI surveillance after colon surgery with the goal of improving the efficiency of infection control practices by prioritizing patients at high risk.

**Methods:**

We conducted a retrospective study using data from 1508 patients undergoing colon surgery treated between 2018 and 2023 at a single academic medical center. Of these 1508 patients, 66 (4.4%) developed SSIs as adjudicated by infection control practitioners following Centers for Disease Control and Prevention National Healthcare Safety Network criteria. Data included 78 structured variables (eg, demographics, comorbidities, vital signs, laboratory tests, medications, and operative details) and 2 features derived from unstructured clinical notes using natural language processing. ML models<strong>―</strong>logistic regression, random forest, and Extreme Gradient Boosting (XGBoost)<strong>―</strong>were trained using stratified 80/20 train-test splits. Class imbalance was addressed using cost-sensitive learning and the synthetic minority oversampling technique. Model performance was evaluated using precision, recall, *F*_1_-score, area under the receiver operating characteristic curve, and Brier scores for calibration.

**Results:**

Of the 1508 patients, those who developed SSIs had longer hospital stays (mean 8.1, SD 6.8 days vs mean 6.3, SD 10.5 days; *P*<.001), higher rates of an American Society of Anesthesiologists score of 3 (52/66, 79% vs 653/1442, 45.3%; *P*<.001), and elevated white blood cell counts (51/66, 77% vs 734/1442, 50.9%; *P*<.001). XGBoost achieved the best overall performance with an area under the receiver operating characteristic curve of 0.788, precision of 50%, recall of 38%, and Brier score of 0.035. Random forest yielded perfect precision (100%) but lower recall (23%), with a Brier score of 0.034. Logistic regression showed the highest recall (46%) but the lowest precision (10%), with a Brier score of 0.139. Feature importance analysis using Shapley additive explanations (SHAP) values revealed that the top predictors included recovery duration (SHAP=1.18), SSI keyword frequency (SHAP=1.12), patient age (SHAP=1.12), and American Society of Anesthesiologists score (SHAP=0.94), with natural language processing–derived features ranking among the top 10.

**Conclusions:**

ML models can augment traditional SSI surveillance by improving early identification of patients at high risk. The XGBoost model offered the best trade-off between discrimination and calibration, suggesting its utility in clinical workflows. Incorporating structured and unstructured electronic health record data enhances model accuracy and clinical relevance, supporting scalable and efficient infection control practices.

## Introduction

### Background

Surgical site infections (SSIs) are a significant category of health care–associated infections, representing a serious challenge to health care systems worldwide. SSIs are estimated to account for nearly 20% of all health care–associated infections among hospitalized patients [1]. In the United States, SSIs occur in approximately 2% to 4% of patients undergoing inpatient surgical procedures [2]. SSIs can lead to severe complications, including increased morbidity, extended hospital stays, higher readmission rates, and increased health care costs, ultimately impacting patient outcomes and putting a strain on health care resources [3-5]. The financial burden of SSIs is substantial, with associated costs reaching billions of dollars annually. SSIs are the third most costly type of health care–acquired infection, with an estimated cost of US $20,785 per patient case [6]. Patients with SSIs have a mortality rate twice that of patients without infections [7].

The current surveillance process for SSIs is resource intensive, requiring manual chart reviews by infection control practitioners to monitor surgical procedures and screen for potential infections. This manual surveillance is time-consuming and labor intensive and detracts from direct patient care [8]. The Centers for Disease Control and Prevention (CDC) National Healthcare Safety Network (NHSN) has established comprehensive guidelines to support the systematic tracking and identification of SSIs; yet, the manual workload remains significant [9].

Several studies have applied machine learning (ML) and natural language processing (NLP) to improve the detection and prediction of SSIs. These approaches have shown promise in automating surveillance and improving accuracy by using structured and unstructured clinical data. However, previous research has largely focused on retrospective detection rather than prospective prediction, relied on limited data sources, or failed to integrate both structured and unstructured data [10-13].

### Objectives

To address these limitations, our study introduces an ML-based surveillance tool designed to enhance SSI monitoring. By integrating structured data and clinical notes, our approach improves detection efficiency and accuracy. Automating surveillance through ML reduces the burden on infection control practitioners, allowing them to focus on patients at high risk and critical tasks, ultimately improving patient outcomes and optimizing resource allocation.

## Methods

### Ethical Considerations

This study was reviewed by the University of Massachusetts Chan Medical School (UMass Chan) Institutional Review Board and deemed exempt under institutional guidelines for quality improvement. As a retrospective analysis of existing electronic health record (EHR) data with no patient contact, informed consent was not required. The institutional review board confirmed that secondary use of clinical data did not necessitate additional consent. All data were deidentified in accordance with HIPAA (Health Insurance Portability and Accountability Act) and analyzed within a secure, access-controlled environment at UMass Chan. Only authorized personnel had access to the data. No identifiable patient information appears in images or supplementary materials; all figures are fully anonymized.

### Data Extraction and Preparation

Data for this study were extracted from the UMass Chan Data Lake, which is a copy of the Epic Clarity EHR system at the hospital and refreshes weekly. This data repository comprises 2.5 million unique patients since November 2017. We focused on patients who underwent colon surgeries between 2018 and 2023, identified using Current Procedural Terminology (CPT) codes according to the NHSN guidelines. Our cohort included 1508 procedures, with 66 (4.4%) confirmed SSI cases.

### Cohort Inclusion and Exclusion Criteria

We identified all adult patients (aged ≥18 years) who underwent colon surgery at the University of Massachusetts Memorial Medical Center between January 1, 2018, and December 31, 2023, using CPT procedure codes per NHSN guidelines. Patients were included if they were aged ≥18 years at the time of surgery, underwent elective or urgent colon resection procedures (CPT codes 44140-44160 and 44204-44208), and had available postoperative follow-up data for at least 30 days. We excluded patients with noncolon procedures or combined multiorgan resections, those with missing critical EHR data (eg, American Society of Anesthesiologists [ASA] score, surgery date, or outcome label), and patients who were deceased before completion of the 30-day postoperative surveillance window. After applying these criteria, 1508 unique procedures remained for analysis.

### Outcome Labeling and Gold Standard

All SSI labels were assigned by the hospital’s infection prevention and control (IPC) team according to CDC NHSN criteria. Each postoperative patient who underwent colon surgery is manually reviewed daily by IPC specialists, who screen charts, microbiology reports, wound assessments, and nursing notes against the NHSN definitions. Ambiguous cases are flagged for discussion at a weekly consensus meeting with at least 2 IPC specialists and a supervising epidemiologist, and final SSI determinations are made through consensus. The IPC department also conducts monthly peer audit exercises to ensure consistency with NHSN standards.

To ensure clinical relevance, infection control nurses provided input during the term selection process for clinical note analysis and validated the model’s outputs against real-world clinical scenarios. The dataset included 78 structured variables, such as demographics, medications, laboratory test results, and medical histories (Table 1). In addition, 2 variables—SSI keyword count and SSI negation term count—were derived from unstructured clinical notes using NLP techniques.

**Table 1:** Candidate predictors used to train machine learning models in a retrospective cohort study of postoperative surgical site infection (SSI) after colon surgery at the University of Massachusetts Memorial Medical Center (Worcester, Massachusetts, United States) from January 1, 2018, to December 31, 2023.

Data domain	Data points
Demographics	Gender, race, ethnicity, age, alcohol usage and smoking status
Comorbidities	Obesity, cancer, diabetes, immunological disease, depression, dementia, anemia, heart failure, AIDS, and alcohol
Encounters	Total stay days and inpatient stay
Laboratory tests	Hemoglobin, culture, white blood cell count, and C-reactive protein
Medications	Antibiotics, immunosuppressants, and steroids
Patient	Patient ID and patient encounter ID
Surgery details	Surgery class, surgery procedure code, physician ID, number of procedures, surgery duration, surgery recovery duration, department, room number, wound status, anesthesia type, ASA^a^ score, incision closure, and SSI
Vital signs	BMI
NLP^b^-derived features	SSI_Keyword and SSI_Negation
SSI keywords	“Fever,” “nausea,” “vomiting,” “pain,” “tenderness,” “odynophagia,” “dysphagia,” “hypotension,” “jaundice,” “dysuria,” “abscess,” and “infection”

^a^ASA: American Society of Anesthesiologists.

^b^NLP: natural language processing.

### Unstructured Data Processing

Specifically, we used the spaCy library (Explosion AI) [14] for tokenization and named entity recognition to identify relevant keywords associated with SSIs, such as “redness,” “swelling,” “drainage,” and “purulent discharge.” Negation detection was conducted using the NegEx algorithm [15], which allowed us to determine whether an identified keyword was negated in the context of the clinical note. For example, “no signs of infection” or “denies fever” would be identified as negated terms. These NLP tools enabled us to accurately quantify the occurrence of SSI-related terms and their context within the notes.

Infection-related terms were first extracted from the CDC NHSN SSI surveillance criteria and then reviewed and finalized by IPC nurses—who conduct daily chart surveillance—to ensure relevance to our colon surgery population. No formal statistical testing was conducted on the term list. All SSI related keywords were curated and validated by the IPC nurses based on daily practice. While transformer-based embeddings (eg, BioBERT) may capture richer linguistic patterns, we deferred their use because our IT department could not approve deployment of large language models in the current infrastructure. These contextual methods will be explored in follow-up work.

### Data Processing and Feature Engineering

Data preprocessing involved imputing missing values in numerical columns using column-wise means, one-hot encoding categorical variables, and validating data types for compatibility with ML algorithms. The dataset was processed using the Python pandas library [16] and then deployed to a secure workspace, the Platform for Learning Health System environment at UMass Chan, for further analysis [17].

After median imputation of numerical variables and one-hot encoding of categorical variables, the dataset expanded to 150 features in total: the original 78 numerical inputs, 2 NLP-derived counts, and 70 dummy variables from one-hot encoding. For the logistic regression model, we then generated all second-degree polynomial interaction terms, yielding 11,325 features in the final matrix used for model fitting.

To better understand the relative impact of different imbalance-handling techniques, we conducted an ablation study on all 3 models (logistic regression, random forest, and Extreme Gradient Boosting [XGBoost]). Three strategies were compared: cost-sensitive approach only by applying class weights (class_weight=*balanced* for tree and linear models and scale_pos_weight for XGBoost), oversampling only via 1:1 random upsampling of the minority (SSI) class in the training set, and oversampling combined with cost-sensitive approaches. Pipelines for each strategy were retrained and evaluated on the same held-out test set (n=302), and metrics including precision, recall, *F*_1_-score (for the SSI class), area under the receiver operating characteristic curve (AUC-ROC), and Brier score were recorded.

### Model Development

The dataset was split into training (80%) and validation (20%) sets using stratified sampling to maintain the class distribution. To address class imbalance, we applied cost-sensitive learning [18] and the synthetic minority oversampling technique (SMOTE) [19]. SMOTE works by creating synthetic examples of the minority class by interpolating between existing instances, thereby balancing the class distribution in the training set.

We developed 3 ML models: logistic regression [20], random forest [21], and XGBoost [22]. Each model was chosen based on specific strengths—logistic regression for interpretability, random forest for handling complex interactions, and XGBoost for efficient structured data analysis.

Logistic regression was chosen due to its simplicity and interpretability, which allows health care professionals to understand the relationships between features and outcomes, making it useful for clinical decision-making. Random forest was selected for its ability to handle complex feature interactions and its robustness in managing missing data, making it effective for capturing nonlinear patterns in the dataset [23]. XGBoost was included for its high predictive performance and efficiency, particularly with structured datasets, and its ability to handle imbalanced data effectively through boosting techniques.

For logistic regression, we used L2 regularization [24] to prevent overfitting and applied polynomial features (adding interaction terms between features) to capture potential nonlinear relationships in the data. For random forest, we optimized the number of trees, maximum depth, and minimum samples per leaf to ensure robustness. We used the same feature set for all models, including the polynomial features, to ensure a fair comparison. For XGBoost, we tuned hyperparameters such as learning rate, maximum depth, and regularization parameters through grid search using cross-validation [25].

### Model Calibration

To assess the reliability of each model’s predicted probabilities in a clinical context, we conducted both quantitative and visual calibration analyses on the held-out test set. We computed the Brier score (using scikit-learn’s [Google Summer of Code] brier_score_loss function), which measures the mean squared difference between predicted probabilities and observed outcomes (lower=better calibration). We also generated calibration (reliability) plots via scikit-learn’s calibration_curve function, dividing predictions into 10 equal-width bins, and for each bin, we plotted the mean predicted probability against the observed SSI rate, overlaying the 45° line to represent perfect calibration. All calibration calculations were conducted in Python (version 3.8; Python Software Foundation) using scikit-learn (version 1.0).

### Model Evaluation

Because SSIs represent only approximately 4% of cases, overall accuracy can be misleading. Therefore, we report precision, recall (sensitivity), *F*_1_-score, and AUC-ROC as our primary performance metrics for the minority class, with accuracy included only for completeness.

To assess performance stability, we performed 5-fold stratified cross-validation on the training set and report each metric’s mean and SD.

Performance metrics, including precision, sensitivity (recall), specificity, *F*_1_-score, and AUC-ROC, were calculated to comprehensively evaluate model performance, especially given the imbalanced nature of the dataset. Sensitivity and specificity help assess the model’s ability to correctly identify positive and negative cases, respectively, whereas the AUC-ROC offers an overall measure of discriminative performance. The final models were validated on the reserved 20% of the dataset to assess their generalizability.

## Results

### Study Population and Characteristics

A total of 1508 patients met the inclusion criteria (n=66, 4.4% with SSIs and n=1442, 95.6% without SSIs). Table 2 presents the comprehensive characteristics of the study population comparing patients with and without SSIs. The table is organized into demographic, clinical, surgical, and medication-related variables to facilitate interpretation (Multimedia Appendix 1).

Demographically, patients with SSIs were significantly older (mean age 61.1, SD 8.9 years, *P*=.01) than those without SSIs (mean age 58.5, SD 15.4 years), with most SSI cases (38/66, 58%) occurring in the category of 61 to 80 years. A higher proportion of female individuals developed SSIs (43/66, 65% vs 765/1442, 53.1% in the non-SSI group), although this difference did not reach statistical significance (*P*=.07).

The ASA score [26] differed significantly between groups (*P*<.001), with a substantially higher percentage of patients with SSIs having an ASA score of 3 (52/66, 79% vs 653/1442, 45.3%), indicating greater preoperative risk and comorbidity burden. Patients who developed SSIs showed a higher prevalence of several comorbidities, including diabetes (17/66, 26% vs 265/1442, 18.4%; *P*=.18), depression (17/66, 26% vs 296/1442, 20.5%; *P*=.18), anemia (24/66, 36% vs 423/1442, 29.3%; *P*=.28), hypertension (36/66, 55% vs 655/1442, 45.4%; *P*=.18), and chronic kidney disease (9/66, 14% vs 123/1442, 8.5%; *P*=.23), although these differences individually did not reach statistical significance.

Surgical and procedural characteristics revealed important differences. Patients who developed SSIs underwent longer surgeries (mean duration 262.5, SD 137.3 min vs 229.9, SD 103.6 min), had a significantly higher number of procedures (mean 1.7, SD 1.1 vs 1.4, SD 0.7; *P*=.02), and experienced substantially longer hospital stays (mean 8.1, SD 6.8 days vs 6.3, SD 10.5 days; *P*<.001). Wound classification showed significant variation, with contaminated wounds being more common in the SSI group (27/66, 41% vs 397/1442, 27.5%; *P*=.03) and clean-contaminated wounds being less common (24/66, 36% vs 799/1442, 55.4%; *P*=.004).

Laboratory and medication factors also demonstrated significant associations with SSI development. Abnormal white blood cell (WBC) counts were significantly more common in patients with SSIs (51/66, 77% vs 734/1442, 50.9%; *P*<.001), defined as WBC counts of >11 before surgery. Steroid use, categorized as receiving any steroid within 12 months before surgery, was significantly higher in the SSI group (55/66, 83% vs 846/1442, 58.7%; *P*<.001). All patients who developed SSIs had received antibiotics.

**Table 2:** Bivariate comparisons of patient, operative, laboratory, and medication characteristics by 30-day National Healthcare Safety Network–defined surgical site infection (SSI) status in a retrospective single-center cohort of patients undergoing colon surgery at the University of Massachusetts Memorial Medical Center (Worcester, Massachusetts, United States) from 2018 to 2023. Only variables with *P*<.05 are shown.

Variable	No SSI (n=1442)	SSI (n=66)	*P* value
Age (y), mean (SD)	58.5 (15.4)	61.1 (8.9)	.01
ASA^a^ score of 3, n (%)	653 (45.3)	52 (78.8)	<.001
Contaminated wound, n (%)	397 (27.5)	27 (40.9)	.03
Length of stay (d), mean (SD)	6.3 (10.5)	8.1 (6.8)	<.001
WBC^b^ flag—high, n (%)	734 (50.9)	51 (77.3)	<.001
Steroid use, n (%)	846 (58.7)	55 (83.3)	<.001

^a^ASA: American Society of Anesthesiologists.

^b^WBC: white blood cell.

### Imbalance Handling and Ablation Study Results

To evaluate the effectiveness of different approaches for handling class imbalance in our dataset, we performed an ablation study that compares three strategies: cost-sensitive learning, random oversampling, and a combination of both techniques. Each strategy was applied to all three ML models (logistic regression, random forest, and XGBoost) and evaluated on the same held-out set to ensure fair comparison. The results of this ablation study are presented in Table 3, which shows the performance metrics for each model and strategy combination.

**Table 3:** Ablation study comparing class imbalance–handling strategies—cost-sensitive learning, random oversampling, and their combination—for predicting 30-day National Healthcare Safety Network–defined surgical site infections after colon surgery in a retrospective single-center cohort (University of Massachusetts Memorial Medical Center, Worcester, Massachusetts, United States; 2018-2023).

Model and strategy		Precision (%)	Recall (%)	*F*_1_-score	AUC-ROC^a^	Brier score
**Logistic regression**						
	Cost-sensitive learning	9.84	46.15	0.162	0.709	0.143
	Oversampling only	10.34	46.15	0.169	0.704	0.140
	Oversampling+cost-sensitive learning	10.34	46.15	0.169	0.704	0.140
**Random forest**						
	Cost-sensitive learning	100	15.38	0.267	0.776	0.032
	Oversampling only	100	15.38	0.267	0.777	0.031
	Oversampling+cost-sensitive learning	100	15.38	0.267	0.777	0.031
**XGBoost^b^**						
	Cost-sensitive learning	57.14	30.77	0.400	0.719	0.036
	Oversampling only	41.67	38.46	0.400	0.758	0.043
	Oversampling+cost-sensitive learning	41.67	38.46	0.400	0.758	0.043

^a^AUC-ROC: area under the receiver operating characteristic curve.

^b^XGBoost: Extreme Gradient Boosting.

### Model Performance and Calibration

**Figure 1:**
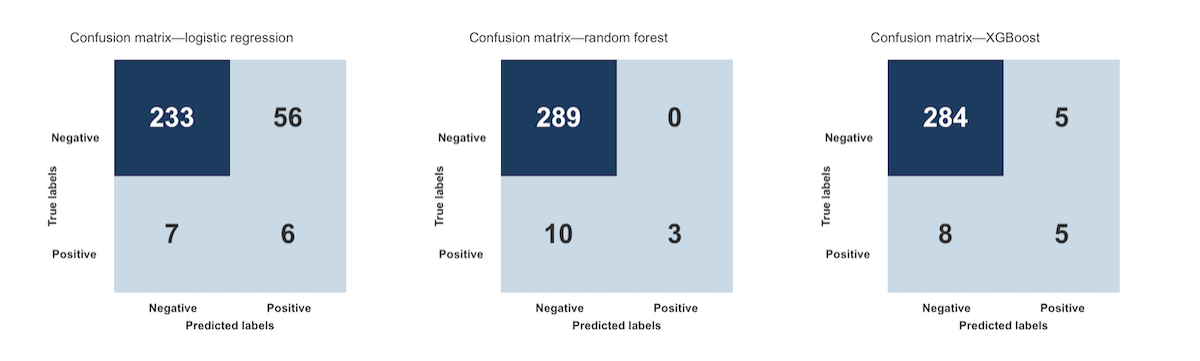
Confusion matrices on the 20% held-out test set for the logistic regression, random forest, and Extreme Gradient Boosting (XGBoost) models predicting 30 day National Healthcare Safety Network–defined surgical site infections (SSIs) after colon surgery in a retrospective single-center cohort (University of Massachusetts Memorial Medical Center, Worcester, Massachusetts, United States; 2018-2023; N=1508 with 66/1508, 4.4% SSIs).

Table 4 summarizes each model’s precision, recall, *F*_1_-score, and AUC-ROC for SSI detection as means and SDs from 5-fold cross-validation alongside Brier scores on the held-out test set. Accuracy was high for all models but less informative given the 4.4% (66/1508) SSI rate. Confusion matirces for all three models on the held-out test are shown in Figure 1.

**Figure 2:**
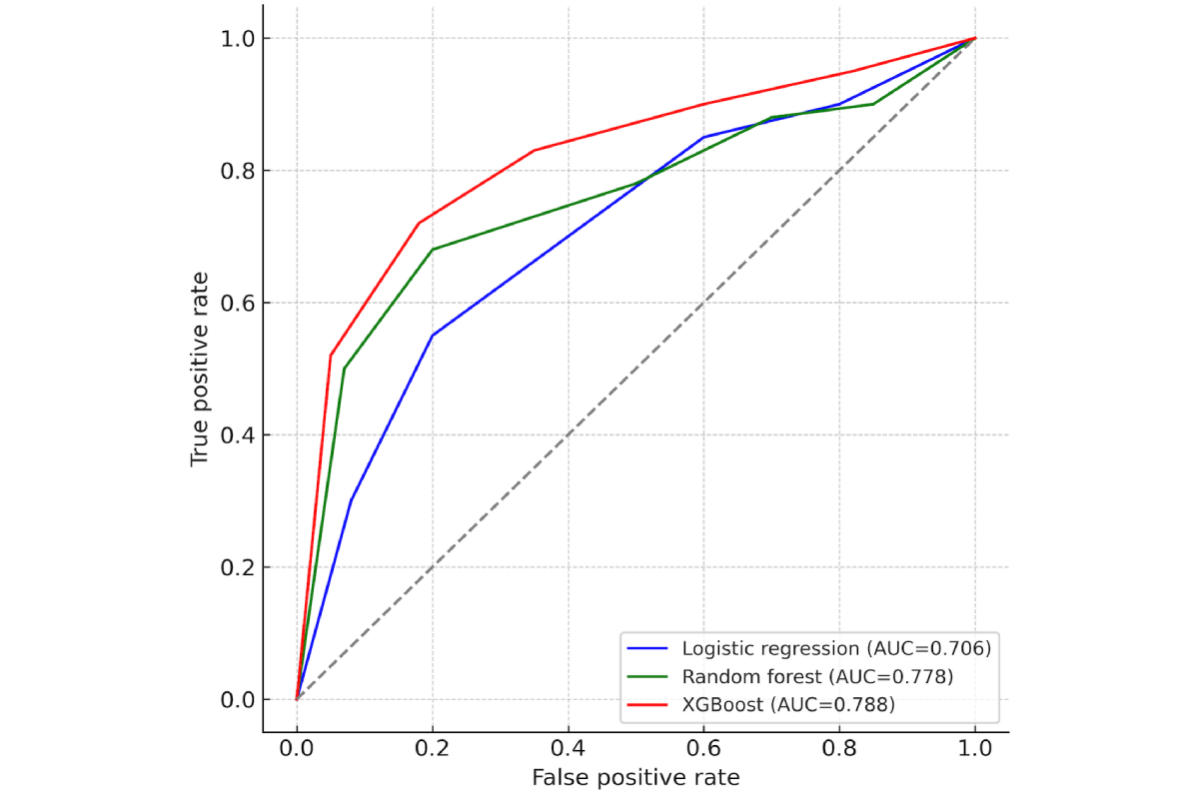
Receiver operating characteristic (ROC) curves on the 20% held-out test set for the logistic regression, random forest, and Extreme Gradient Boosting (XGBoost) models trained to predict 30-day National Healthcare Safety Network–defined surgical site infections following colon surgery in a retrospective single center cohort (University of Massachusetts Memorial Medical Center, Worcester, Massachusetts, United States; 2018-2023). Area under the ROC curve values summarize discrimination. AUC: area under the curve.

**Figure 3:**
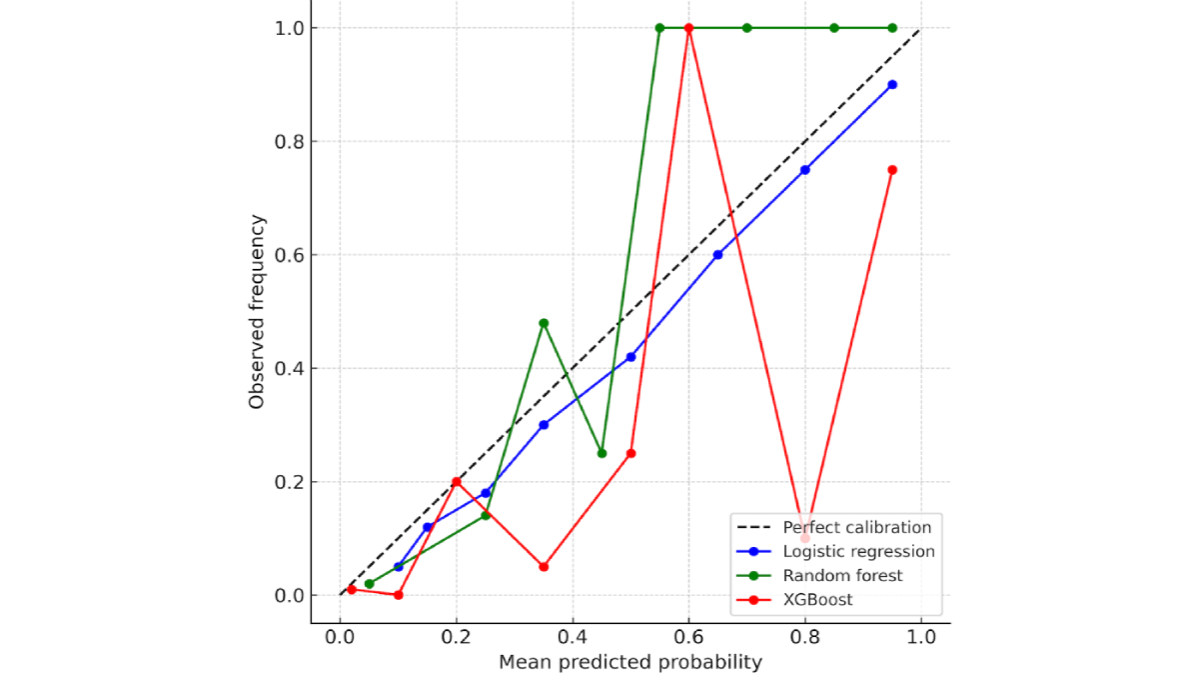
Calibration (reliability) curves on the 20% held-out test set for logistic regression, random forest, and Extreme Gradient Boosting (XGBoost) predicting 30-day National Healthcare Safety Network–defined surgical site infections after colon surgery in a retrospective single-center cohort (University of Massachusetts Memorial Medical Center, Worcester, Massachusetts, United States; 2018-2023). Predictions were binned into 10 equal-width bins; the dashed 45° line indicates perfect calibration.

In terms of discrimination, XGBoost achieved the highest AUC-ROC (0.788), followed by random forest (0.778) and logistic regression (0.706; Figure 2). Regarding calibration, random forest and XGBoost both exhibited low Brier scores (0.034 and 0.035, respectively), indicating well-calibrated probability estimates, whereas logistic regression’s higher Brier score (0.139) reflects moderate miscalibration (Figure 3).

Among the 3 models, XGBoost demonstrated the highest AUC-ROC score (0.788) and *F*_1_-score (0.43), suggesting the best overall discriminative ability and balance between precision and recall. The random forest model showed perfect precision but lower recall, indicating that it was highly conservative in predicting SSIs. Logistic regression had the highest recall but the lowest precision, suggesting that it was more liberal in flagging potential SSI cases but at the cost of many false positives.

**Table 4:** Model performance for predicting 30-day National Healthcare Safety Network–defined surgical site infections after colon surgery in a retrospective single-center cohort (University of Massachusetts Memorial Medical Center, Worcester, Massachusetts, United States; 2018-2023)^a^.

Model	Precision (%), mean (SD)	Recall (%), mean (SD)	*F*_1_-score, mean (SD)	AUC-ROC^b^, mean (SD)	Brier score
Logistic regression	10.7 (3.9)	56.0 (18.5)	0.18 (0.06)	0.775 (0.059)	0.139
Random forest	80.0 (40.0)	11.1 (6.8)	0.19 (0.11)	0.756 (0.046)	0.034
XGBoost^c^	40.0 (17.0)	20.5 (8.6)	0.27 (0.11)	0.735 (0.059)	0.035

^a^Five-fold stratified cross-validation results are shown; Brier scores were computed on the 20% held-out test set.

^b^AUC-ROC: area under the receiver operating characteristic curve.

^c^XGBoost: Extreme Gradient Boosting.

### Statistical Comparison of Area Under the Curve

To formally assess whether observed area under the curve (AUC) differences were significant, we conducted a paired bootstrap analysis with 1000 resamples of the test set. The 95% CIs and *P* values are shown in Table 5. All intervals overlapped, and both pairwise *P* values exceeded .05, indicating no statistically significant differences between XGBoost and the other models.

Because all intervals overlapped substantially and both *P* values exceeded .05, we conclude that the small observed AUC differences were not statistically significant. These results support our interpretation that XGBoost and random forest performed equivalently in discriminating SSI risk and that XGBoost’s edge over logistic regression did not reach significance under bootstrap testing.

**Table 5:** Statistical comparison of model areas under the curve (AUCs) via paired bootstrap (1000 resamples) on the 20% held-out test set in a retrospective single-center study predicting 30-day National Healthcare Safety Network–defined surgical site infections after colon surgery (University of Massachusetts Memorial Medical Center, Worcester, Massachusetts, United States; 2018-2023).

	AUC (95% CI)	*P* value
LR^a^	0.706 (0.538-0.850)	—^b^
RF^c^	0.778 (0.588-0.935)	—
XGB^d^	0.788 (0.607-0.933)	—
XGB vs LR	—	.50
XGB vs RF	—	.79

^a^LR: logistic regression.

^b^Not applicable.

^c^RF: random forest.

^d^XGB: Extreme Gradient Boosting.

### Feature Importance (Shapley Additive Explanations Analysis)

Table 6 lists the top 10 features by mean absolute Shapley additive explanations (SHAP) value for the XGBoost model, demonstrating the influence of both structured EHR variables and our NLP-derived metrics on SSI risk prediction.

Feature importance analysis revealed that the most predictive factors for SSI risk included ASA score, patient age, wound classification (particularly contaminated wounds), steroid use, and laboratory indicators such as WBC count. The NLP-derived features (SSI keyword count and negation term count) also contributed significantly to the models’ predictive performance, highlighting the value of incorporating unstructured clinical notes in SSI risk assessment. Notably, ssi_keyword (rank 2) and ssi_negated (rank 7) were among the top predictors—on par with established clinical features such as age and ASA score.

**Table 6:** Top 10 predictors of 30-day National Healthcare Safety Network–defined surgical site infections (SSIs) after colon surgery based on mean absolute Shapley additive explanations (SHAP) values from the final Extreme Gradient Boosting model trained on the retrospective single-center cohort (University of Massachusetts Memorial Medical Center, Worcester, Massachusetts, United States; 2018-2023)a.

Rank	Feature	SHAP value, mean (SD)
1	surgery_minutes_in_recovery	1.1761
2	ssi_keyword	1.1217
3	surgery_patient_age	1.1188
4	asa_score	0.9354
5	surgery_minutes_in_or	0.7716
6	average_bmi	0.7397
7	ssi_negated	0.7311
8	surgery_physician_id_E8779	0.4223
9	length_of_stay	0.3408
10	patient_gender_female	0.3100

^a^Predictors include structured electronic health record features (eg, American Society of Anesthesiologists score, operative times, BMI, and length of stay) and natural language processing–derived features from clinical notes (SSI keyword and negation counts).

## Discussion

### Principal Findings

This study aimed to develop and evaluate ML models for the early prediction of SSIs following colon surgery using both structured EHR data and unstructured clinical notes. Our main findings show that ML models, particularly XGBoost, can effectively augment traditional surveillance practices by providing well-calibrated, discriminative risk predictions that prioritize patients at high risk. Among the models tested, XGBoost demonstrated the best balance between precision and recall (AUC-ROC=0.788; precision=50%; recall=38%), whereas random forest achieved perfect precision at the cost of low sensitivity (recall=23%).

A more detailed analysis reveals that each model has specific strengths that may suit different clinical priorities. The XGBoost model provides a practical compromise between sensitivity and specificity, which is ideal for resource-limited infection control teams aiming to balance workload and risk. Random forest’s high precision makes it suitable for contexts in which false positives must be minimized, whereas logistic regression offers higher recall but risks overburdening staff due to its lower precision. Our findings are consistent with those of previous literature in emphasizing the importance of model calibration for real-world implementation; the low Brier scores for both XGBoost and random forest suggest reliable probability estimates that can guide clinical triage. Furthermore, the integration of NLP-derived features—such as SSI keyword frequency and negation detection—significantly improved predictive performance, with these variables ranking among the top 10 predictors by SHAP value. This supports previous work [27,28] on the added value of unstructured clinical data in infection surveillance, but our study differs by using these data prospectively—leveraging information available before infection onset—rather than relying on retrospective documentation after SSIs have already occurred.

Previous studies have also demonstrated strong performance in SSI identification using ML but with important methodological differences. One study using NLP achieved a sensitivity and positive predictive value of 97% for SSI detection [29] but relied on postinfection clinical notes, limiting its utility for early intervention. Another study applied logistic regression and tree-based models to preoperative blood test results, achieving an AUC of 86% [30], although it used a more balanced dataset and focused narrowly on laboratory values. In contrast, our approach emphasizes prospective prediction using only preinfection data and incorporates a broader set of features—including operative and demographic characteristics and unstructured clinical text—to support earlier, more comprehensive risk assessment and real-time clinical deployment. This multimodal integration increases the generalizability and interpretability of our model, making it better suited for prospective deployment within existing clinical workflows.

To support clinical adoption, we propose embedding the model’s SSI risk scores directly into the EHR’s surveillance dashboard. Each morning, the model generates risk scores for all patients undergoing colon surgery, automatically flagging those above a configurable threshold (eg, the top 10%). Flagged patients appear on an IPC nurse worklist for prioritized chart review, focusing on vital signs, wound assessments, and microbiology. The threshold can be adjusted based on operational capacity—for example, flagging the top 5% during busy periods or expanding to 15% during lower volume. Nurse adjudications (SSI vs no SSI) are fed back into the system to support model retraining and recalibration over time. This workflow focuses human effort on high-risk cases, enhances surveillance efficiency, and remains adaptable to fluctuating staffing or patient volumes.

### Limitations

Our evaluation showed that SMOTE was more effective than cost-sensitive learning in improving model performance for the minority class, increasing recall by approximately 15% while maintaining similar precision. However, both approaches still exhibited some bias toward the majority class, particularly the random forest model. The severe class imbalance in our dataset (only 66/1508, 4.4% were SSI cases) presents a significant challenge for model development and evaluation. Although we used techniques to address this imbalance, the models’ performance in identifying positive cases remained suboptimal, as evidenced by the modest recall values. Future work could explore advanced synthetic generation methods [31] to address severe class imbalance.

Due to the severe class imbalance, feature importance scores—particularly from tree-based models—may be driven primarily by patterns in the majority (non-SSI) class. Although we applied both SMOTE and cost-sensitive learning to mitigate this imbalance before model training, readers should interpret importance rankings with caution. In future work, we plan to explore class-specific importance measures (eg, SHAP values stratified by outcome) to obtain a more balanced view of predictors for the minority class.

In addition, the use of retrospective data from a single health care center may introduce biases related to specific patient populations and clinical practices, potentially limiting the generalizability of the results. The predictive factors identified in our specific setting might not hold the same importance in other health care environments with different patient demographics, surgical practices, or infection control protocols.

Furthermore, our approach to processing unstructured clinical notes was limited to keyword counting and negation detection. More sophisticated NLP techniques such as embedding-based methods or transformer models might yield better feature extraction and, ultimately, enhance predictive performance [32-34].

### Conclusions

This study demonstrates that ML models can enhance SSI surveillance by helping clinicians prioritize patients at high risk. Our ML-based tool, which integrates structured EHR data and unstructured clinical notes, offers a scalable approach to improve monitoring efficiency.

Among the models evaluated, XGBoost provided the best balance of precision, recall, and calibration, although each model presents unique strengths suited to different clinical needs. By triaging patients based on predicted risk, the tool can reduce manual workload and support more timely, targeted interventions to improve patient outcomes.

While this pilot study shows proof of concept, broader validation is needed to ensure generalizability and clinical utility. We plan to partner with other institutions using the Observational Medical Outcomes Partnership Common Data Model and federated learning to enable privacy-preserving, cross-site model training. Performance will be assessed through calibration, discrimination, and operational impact metrics.

Beyond technical refinement, this work underscores the potential of artificial intelligence–driven tools to transform infection surveillance from reactive monitoring to proactive, risk-based care. Future efforts should focus on handling class imbalance, improving NLP feature extraction, and ensuring model reliability through post hoc calibration and multisite validation.

Ultimately, this research advances the responsible and scalable integration of artificial intelligence into clinical workflows to support more targeted, efficient infection prevention.

## Appendix

Multimedia Appendix 1 Detailed cohort characteristics and model performance metrics, including confusion matrices and receiver operating characteristic curve plots for each machine learning model.

## Abbreviations

ASA: American Society of Anesthesiologists
AUC: area under the curve
AUC-ROC: area under the receiver operating characteristic curve
CDC: Centers for Disease Control and Prevention
CPT: Current Procedural Terminology
EHR: electronic health record
HIPAA: Health Insurance Portability and Accountability Act
IPC: infection prevention and control
ML: machine learning
NHSN: National Healthcare Safety Network
NLP: natural language processing
SHAP: Shapley additive explanations
SMOTE: synthetic minority oversampling technique
SSI: surgical site infection
UMass Chan: University of Massachusetts Chan Medical School
WBC: white blood cell
XGBoost: Extreme Gradient Boosting

## Data Availability

The datasets generated or analyzed during this study are not publicly available due to usage of individual-level electronic health record data that includes protected health information such as structured tables and free-text clinical notes and, therefore, cannot be publicly shared under US HIPAA (Health Insurance Portability and Accountability Act) regulations and the terms of our institutional review board approval. Deidentified, aggregate summary outputs underlying the manuscript (eg, model performance tables) are provided in Multimedia Appendix 1.
