# Supplementary material for: Machine Learning–Enhanced Surveillance for Surgical Site Infections in Patients Undergoing Colon Surgery: Model Development and Evaluation Study
Source: JMIR Form Res. 2025 Oct 1;9:e75121. doi: 10.2196/75121 (PMC12530156; doi:10.2196/75121)
Supplement: Multimedia Appendix 1 [file formative_v9i1e75121_app1.docx]

## **Multimedia Appendix 1**

Detailed cohort characteristics and model performance metrics including confusion matrices and ROC Curve plots for each machine learning model.

**Table 2** (Full characteristics). Demographic, clinical, surgical, laboratory, and medication characteristics for all colon surgery patients in the retrospective cohort (n=1508) at UMass Memorial Medical Center (Worcester, Massachusetts, USA), January 1, 2018-December 31, 2023, stratified by 30‑day NHSN‑defined SSI status (non‑SSI vs SSI).

| **Variable** | **All Patients (n=1508)** | **No SSI (n=1442)** | **SSI (n=66)** | **P-value** |
| --- | --- | --- | --- | --- |
| **Gender** |  |  |  | 0.0717 |
| Female | 808.0 (53.6%) | 765 (53.1%) | 43 (65.2%) |  |
| Male | 700.0 (46.4%) | 677 (46.9%) | 23 (34.8%) |  |
| **Race** |  |  |  | 0.5682 |
| White | 1323.0 (87.7%) | 1262 (87.5%) | 61 (92.4%) |  |
| Black or African American | 47.0 (3.1%) | 45 (3.1%) | 2 (3.0%) |  |
| Asian | 18.0 (1.2%) | 16 (1.1%) | 2 (3.0%) |  |
| Other | 120.0 (8.0%) | 119 (8.3%) | 1 (1.6%) |  |
| Ethnicity |  |  |  | 0.4252 |
| Not Hispanic or Latino | 1379.0 (91.4%) | 1315 (91.2%) | 64 (97.0%) |  |
| Hispanic or Latino | 114.0 (7.6%) | 112 (7.8%) | 2 (3.0%) |  |
| Decline/Unknown | 15.0 (1.0%) | 15 (1.0%) | 0 (0.0%) |  |
| **Age Category** |  |  |  | 0.0146 |
| <18 | 15.0 (1.0%) | 15 (1.0%) | 0 (0.0%) |  |
| 18-40 | 145.0 (9.6%) | 144 (10.0%) | 1 (1.5%) |  |
| 41-60 | 605.0 (40.1%) | 578 (40.3%) | 27 (40.9%) |  |
| 61-80 | 653.0 (43.3%) | 615 (42.9%) | 38 (57.6%) |  |
| >80 | 83.0 (5.5%) | 83 (5.8%) | 0 (0.0%) |  |
| Age (years) | Mean: 58.6 ± 15.2 | Mean: 58.5 ± 15.4 | Mean: 61.1 ± 8.9 | 0.2589 |
| **BMI Category** |  |  |  | 0.0681 |
| Underweight | 47.0 (3.1%) | 47 (3.3%) | 0 (0.0%) |  |
| Normal | 426.0 (28.2%) | 404 (28.4%) | 22 (33.3%) |  |
| Overweight | 509.0 (33.8%) | 488 (34.3%) | 21 (31.8%) |  |
| Obese I | 306.0 (20.3%) | 295 (20.7%) | 11 (16.7%) |  |
| Obese II | 150.0 (9.9%) | 138 (9.7%) | 12 (18.2%) |  |
| Obese III | 52.0 (3.4%) | 52 (3.7%) | 0 (0.0%) |  |
| BMI (kg/m²) | Mean: 28.4 ± 6.8 | Mean: 28.4 ± 6.9 | Mean: 28.9 ± 5.8 | 0.5055 |
| **ASA Score** |  |  |  | 0.0000 |
| 1 | 14.0 (0.9%) | 14 (1.0%) | 0 (0.0%) |  |
| 2 | 756.0 (50.1%) | 743 (51.5%) | 13 (19.7%) |  |
| 3 | 705.0 (46.8%) | 653 (45.3%) | 52 (78.8%) |  |
| 4-5 | 33.0 (2.2%) | 32 (2.2%) | 1 (1.5%) |  |
| **Comorbidities** |  |  |  |  |
| Number of Comorbidities | Mean: 1.9 ± 1.7 | Mean: 1.9 ± 1.7 | Mean: 2.2 ± 1.8 | 0.0961 |
| Obesity | 325.0 (21.6%) | 308 (21.4%) | 17 (25.8%) | 0.4860 |
| Cancer | 287.0 (19.0%) | 276 (19.1%) | 11 (16.7%) | 0.7337 |
| Diabetes | 282.0 (18.7%) | 265 (18.4%) | 17 (25.8%) | 0.1795 |
| Depression | 313.0 (20.8%) | 296 (20.5%) | 17 (25.8%) | 0.3846 |
| Anemia | 447.0 (29.6%) | 423 (29.3%) | 24 (36.4%) | 0.2779 |
| Heart Failure | 59.0 (3.9%) | 57 (4.0%) | 2 (3.0%) | 1.0000 |
| Hypertension | 691.0 (45.8%) | 655 (45.4%) | 36 (54.5%) | 0.1841 |
| Chronic Kidney Disease | 132.0 (8.8%) | 123 (8.5%) | 9 (13.6%) | 0.2252 |
| Chronic Liver Disease | 5.0 (0.3%) | 4 (0.3%) | 1 (1.5%) | 0.2007 |
| Surgical Characteristics |  |  |  |  |
| Surgery Duration (minutes) | Mean: 231.3 ± 105.5 | Mean: 229.9 ± 103.6 | Mean: 262.5 ± 137.3 | 0.1400 |
| Number of Procedures | Mean: 1.4 ± 0.8 | Mean: 1.4 ± 0.7 | Mean: 1.7 ± 1.1 | 0.0216 |
| Length of Stay (days) | Mean: 6.4 ± 10.4 | Mean: 6.3 ± 10.5 | Mean: 8.1 ± 6.8 | 0.0004 |
| Inpatient Surgery | 231.0 (15.3%) | 220 (15.3%) | 11 (16.7%) | 0.8916 |
| **Wound Classification** |  |  |  |  |
| Clean Wound | 121.0 (8.0%) | 115 (8.0%) | 6 (9.1%) | 0.9246 |
| Clean-Contaminated Wound | 823.0 (54.6%) | 799 (55.4%) | 24 (36.4%) | 0.0036 |
| Contaminated Wound | 424.0 (28.1%) | 397 (27.5%) | 27 (40.9%) | 0.0261 |
| **Anesthesia** |  |  |  |  |
| General Anesthesia | 1450.0 (96.2%) | 1384 (96.0%) | 66 (100.0%) | 0.1763 |
| General + Epidural | 54.0 (3.6%) | 54 (3.7%) | 0 (0.0%) | 0.1685 |
| **Laboratory Results** |  |  |  |  |
| Hemoglobin Flag | 1503.0 (99.7%) | 1437 (99.7%) | 66 (100.0%) | 1.0000 |
| C-Reactive Protein Flag | 237.0 (15.7%) | 230 (16.0%) | 7 (10.6%) | 0.3204 |
| White Blood Cell Flag | 785.0 (52.1%) | 734 (50.9%) | 51 (77.3%) | 0.0000 |
| Positive Culture | 8.0 (0.5%) | 8 (0.6%) | 0 (0.0%) | 1.0000 |
| **Medications** |  |  |  |  |
| Antibiotic Use | 1467.0 (97.3%) | 1401 (97.2%) | 66 (100.0%) | 0.2563 |
| Immunosuppressive Therapy | 73.0 (4.8%) | 70 (4.9%) | 3 (4.5%) | 1.0000 |
| Steroid Use | 901.0 (59.7%) | 846 (58.7%) | 55 (83.3%) | 0.0001 |
